# Epidemiology of Spinal Cord Injuries and their Outcomes: A Retrospective Study at the King Khalid Hospital

**DOI:** 10.7759/cureus.6511

**Published:** 2019-12-30

**Authors:** Khlood M Mehdar, Ahood A Mahjri, Ali A Al Rashah, Abdullah Alyazidi‏

**Affiliations:** 1 Medicine, Najran University, Najran, SAU; 2 Neurosurgery, Najran University, Najran, SAU; 3 Neurosurgery, King Khalid Hospital, Najran, SAU

**Keywords:** spinal cord injury, road traffic accidents, traumatic spinal cord injury, neurosurgery, paraplegia, quadriplegia, fall, sci, najran, saudi arabia

## Abstract

Background: Spinal cord injury (SCI) is a life-changing neurological injury. Besides having significant implications for the patient, SCI places a considerable burden upon healthcare resources. Common causes of SCI include falls, road traffic accident (RTA), gunshots, and bomb blast. There is limited national data recording the aetiology of SCI in Saudi Arabia. The aim of this study is to collate SCI data obtained from patients admitted to King Khalid hospital (KKH), Najran, over the year covering June 2018 to June 2019.

Aim: To measure the frequency and epidemiology of SCI at KKH for all patients admitted to the hospital during the study period; also to evaluate the aetiologies and use the information to propose strategies to minimise SCI.

Methods: Data for all patients admitted to KKH with SCI were assessed. Reviewed data included patients’ age, gender, nationality, the cause of SCI, and the outcome.

Results: Throughout the study duration, a total of 182 patients were admitted with SCI. Of those, 53% were male, many of whom were between the ages of 16 and 30 years. Amongst males, the most common cause of SCI was RTA (59%); the second most common cause was falls (15%), which is almost tied with bomb blast (15%). Falls are the most common cause of SCI in females (13%); RTAs are the second most common cause of SCI in females. The majority of young patients were stable and had improved. However, six patients were paraplegic following RTA-initiated SCI; four patients were quadriplegic.

Conclusion: The most common cause of SCI is RTA, which is followed by fall and bomb blast. The recovery prospects of young SCI patients tend to be better than the prospects of elderly patients.

## Introduction

The spinal cord (SC) is part of the central nervous system that connects the brain to the rest of the body. Protected by the vertebral column, the SC is the means by which nerve impulses are transmitted between the body and brain [1]. Given the significance of the SC, injuries to it can result in life-changing consequences and disability that not only affect the patient, but also their families [2]. The consequences of spinal cord injury (SCI) include sensory deficits and paralysis, making SCI a major medical issue that puts a considerable burden on the healthcare system [1].

The annual cost of SCI in the United States is estimated to range from 5 to 9 million dollars; in Australia, it is estimated to be approximately 2 billion dollars [3]. The causes of SCI between and within countries are variable. RTAs involving bicycles, pedestrians and vehicles, are the most common causes of SCI. High-risk activities, such as hang gliding, rock climbing, and sailing are also considered as causes of SCI. Such activities can expose the SC to significant forces, potentially leading to SCI. Falls are also another common cause of injury, being responsible for 20% of all SCI; those over the age of 65 years are especially vulnerable to SCI from a fall [4].

Although SCI is uncommon in young children, accounting for 1%-10% of all SCI, the fragility of their SC means they are also vulnerable to SCI. Juvenile spines are delicate because the spine’s muscles are underdeveloped and the ligaments are highly flexible [4]. Whilst there is not much information available about the incidence of SCI in Saudi Arabia, the limited data points to RTAs being the country’s most common cause of SCI - this particularly applies to young people. According to Alshahri et al. (2012), the risk of SCI from an RTA is greatest for males between the ages of 18 and 32 years [4]. This finding is consistent with other studies that report the age group to be most affected by SCI are those between 20 and 30 years of age [4]. In Saudi Arabia, fewer women have been vulnerable to RTA-induced CSI because until recently women were prohibited from driving. However, that edict has now been revoked, so it is probable that there will be an increase in the number of women who experience SCI in Saudi Arabia.

Other countries have collected SCI aetiology, prevalence, treatment and rehabilitation data, but little of this important information is available for Saudi Arabia. Therefore, there is a need to collect and analyse SCI data and outcomes for Saudi Arabia. This descriptive study aims to do just that by quantifying the number of patients admitted for SCI to KKH in Najran city in Saudi Arabia and evaluating the outcomes.

Najran city is in the southern region of Saudi Arabia. The KKH is the only hospital in the region with a neurosurgery clinic. All neurological cases are referred to this hospital, hence its suitability for conducting a study.

## Materials and methods

A descriptive retrospective study was carried out on all patients admitted to the KHH, Najran, SA with SCI during the period of June 2018 to June 2019. Data were collected in a data sheet for all patients admitted in the mentioned period (n=182) and they were classified according to their age group, gender, and nationality (Saudi or Non-Saudi). Also, patients were categorized according to the outcomes of the injury including paraplegia and quadriplegia. Then, data were coded and inserted to excel software. Descriptive statistics were used to present each categorical data as percentages and all data were blotted in bar graphs. Error bar was used to present the standard deviation (SD).

## Results

**Figure 1:**
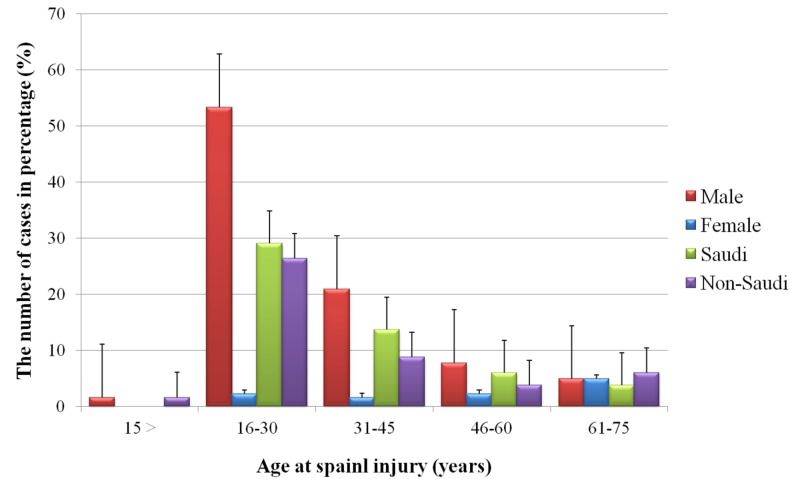
Age at spinal cord injury for patients admitted to the King Khalid Hospital, Najran, Saudi Arabia

Between June 2018 and June 2019, 182 patients were admitted to the KKH neurosurgery department for SCI. Of these cases, only 21 patients were female. Most of the patients fell between the ages of 16 and 30 years (Figure 1).

**Figure 2:**
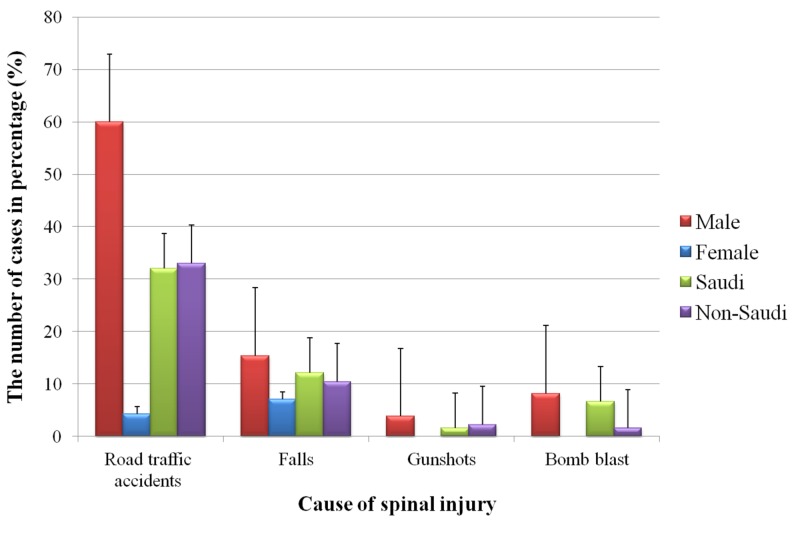
Causes of spinal cord injury for patients admitted to the King Khalid Hospital, Najran, Saudi Arabia

Evaluating the aetiology of SCI, at 64% of all SCI patients, RTAs emerge as the most common cause of injury; of all these RTA-initiated injuries, 87% occurred in males. The second most common cause of SCI were falls, accounting for 22%. Twice as many males were admitted for fall-related SCI as females (15% vs 7%). Eight percent of patients suffered their SCI as a consequence of gunshot, and 4% for bomb blast (Figure 2).

**Figure 3:**
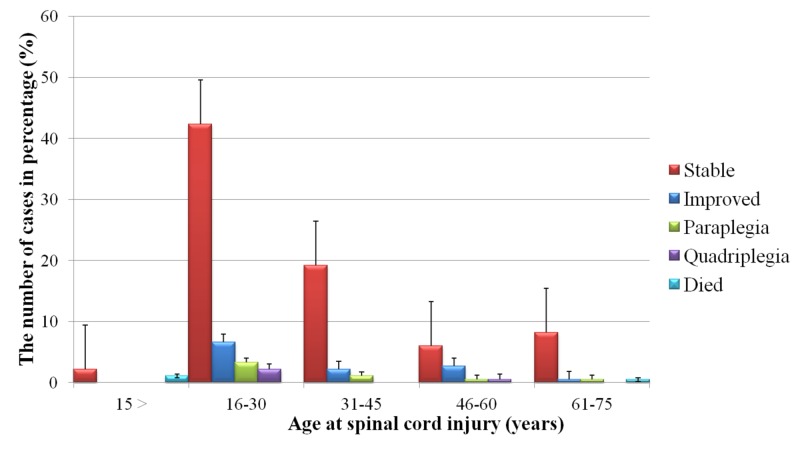
Outcomes of spinal cord injury for each age group of patients admitted to the King Khalid Hospital, Najran, Saudi Arabia

After admission to the hospital, 12% of the SCI patients improved and 78% were stable. Approximately 5% of the patients became paraplegic and 3% quadriplegic; the remaining 2% died (Figure 3).

**Figure 4:**
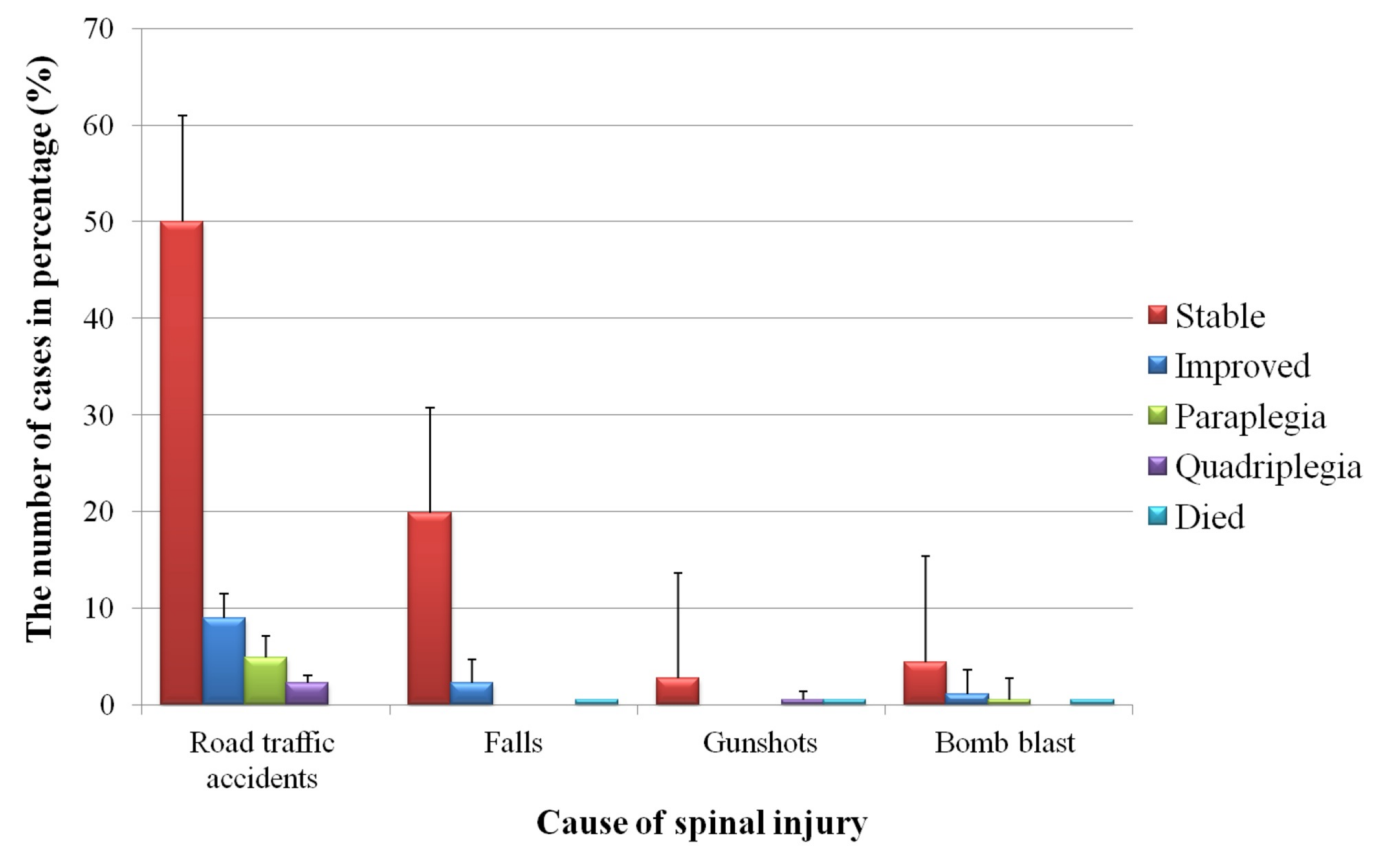
Outcomes of spinal cord injury for each cause of patients admitted to the King Khalid Hospital, Najran, Saudi Arabia

As indicated in Figure 4, of all those patients who had SCI following an RTA, 49% stable and 9% showed improvement. The outcomes following falls were less promising, with only 2% showing improvement and 20% being stable. Two patients had quadriplegia - one as the result of a bomb blast and the other from a gunshot.

## Discussion

SCI is a significant, life-changing, even life-threatening condition commonly causing major disability. Epidemiological data for SCI in Saudi Arabia is limited; therefore, this study set out to establish the incidence of SCI in Najran. A systematic review of SCI in Asian countries found the incidence varied across different countries, ranging from 12 to 62 per million [5]. One study that did determine the incidence in Saudi Arabia found that between 2000 and 2010, it was 38 per million [6]. Based on that result, the incidence in Saudi Arabia is at the higher end of the range of the countries evaluated in the systematic review.

A consistent trend across studies is that the risk of SCI is higher in males than females. For example, the male:female ratio of SCI was 46:1 according to a study undertaken in Riyadh [7]. The result from our study places the male to female ratio at 23:3, which although lower than that reported above, follows the same trend. This also applies to the findings of Alshahri’s study, which had a male:female ratio of 7.5:1 [4]. Recently, a study conducted to assess the Saudi public’s knowledge of SCI showed that the majority of the survey participants were familiar with the basic structures of the SC, while (45.9%) had lacked knowledge about the clinical characteristics of SCI. In particular, young males have insufficient information about SC structure and injuries [8].

The age distribution of SCI patients in our study shows that the prevalence is greatest among those aged between 16 and 30 years. This is consistent with Al-Arabi’s observation that most SCI patients were less than 40 years old [7]. The causes for SCI are diverse, but commonly include bomb blast, gunshot, falls, and RTAs. A retrospective study conducted at the Riyadh military hospital found that of 307 patients with SCI, 85% were the product of RTA; the second most common cause of injury were falls [4]. Another retrospective single-center medical record review revealed that the majority of SCIs were caused by motor vehicle accidents which account for 88.4% among 1128 patients between the period of 2001 to 2016 [9].

Another Saudi Arabian study suggests that the country has one of the world’s highest prevalence for SCI due to RTA [7]. This is consistent with the results of our study, which found 64% of SCI patients admitted to the KKH was as the consequence of an RTA; falls were the second commonest cause of admission for SCI. As already stated, after RTA, falls are the second most common cause of SCI [10]. Of the patients admitted to Hamad Hospital in Qatar with SCI, 36.5% received their injuries through RTAs and 19% from falls [11]. This study found that SCI due to RTA was 25%, compared to 23% caused by falls. Najran city is in close proximity to the border of Yemen, which is at war. Consequently, residents in the city and surrounding areas are more vulnerable than populations in other parts of the country to bomb blasts. Approximately 8% of the SCI patients admitted to KHH acquired their injury through bomb blasts. The risk of disability following SCI varies but can be significant, resulting in paraplegia or quadriplegia [4]. Patients with paraplegic and quadriplegic outcomes were identified in our study, though at incidences much lower than those that were identified in a different Saudi Arabian study. In that study, the incidence of paraplegia was approximately 40%, and more than half of SCI patients had quadriplegia; this contrasts with our results of 6% for paraplegia and 4% for quadriplegia.

## Conclusions

Our study focused on the incidence of SCI in patients admitted to KHH at Najran. Efforts should be made to minimise RTAs in young people and steps taken to prevent SCI occurring in the first place. To prevent SCI, there needs to be a partnership between healthcare workers and educationalists dedicated to promoting awareness of SCI, its primary causes, and outcomes. Attention also needs to be given to methods of preventing SCI.
